# Distributed Secure Economic Dispatch Strategy Based on Robust Graph Theory and W-MSR Algorithm

**DOI:** 10.3390/s25082551

**Published:** 2025-04-17

**Authors:** Jian Le, Jing Wang, Hongke Lang, Weihao Wang

**Affiliations:** 1 School of Electrical Engineering and Automation, Wuhan University, Wuhan 430072, China; whu_wangj@whu.edu.cn (J.W.); langhk@whu.edu.cn (H.L.); 2020302191679@whu.edu.cn (W.W.); 2 Institute of Next Generation Power Systems and International Standards, Wuhan University, Wuhan 430072, China

**Keywords:** distributed economic dispatch, robust networks, W-MSR algorithm, consensus algorithm, information security

## Abstract

The traditional consensus-based distributed economic dispatch strategy may lose system convergency and cause imbalanced power when facing an information attack on the individual power generation unit; thus, it is unable to achieve the dispatching goal. Taking into consideration several kinds of attack behaviors that may exist in a distributed control system, this paper develops models of node attacks from the two aspects of action mode and deployment scope, and analyzes the influence of attack behaviors on the distributed economic dispatch system. Based on the idea of the W-MSR algorithm that deletes the information received from nodes that may be attacked, a distributed security consensus-based economic dispatch strategy is synthetized with the incremental cost of the power generation unit as the consensus variable. Based on the graph robustness index, this paper gives its conditions along with its proof that the communication network topology of the system should be satisfied when adopting the W-MSR algorithm. The simulation results of the IEEE-39 bus distribution network show that the strategy proposed in this paper can effectively counter various information attacks, enhancing both the security and economic efficiency of the distributed economic dispatch system. In addition, the (*F* + 1, *F* + 1)-robust graph is a necessary and sufficient condition to achieve the consensus of the dispatch strategy.

## 1. Introduction

Distributed economic dispatch means that the distributed mode without a dispatch center is used to calculate and adjust the power of each power generation unit under the premise of meeting the basic constraints of the power system to achieve the most economical (usually manifested as the lowest fuel cost) operating mode. Compared with the traditional centralized economic dispatch, the distributed mode has stronger scalability, higher reliability, and more balanced communication and computing burdens, which satisfy the demands of distributed integration and “plug and play” operation, thereby making this mode a research hotspot of current power systems [1,2,3,4].

At present, the research on distributed economic dispatch has made many advances. Its basic idea is to use multi-agent system (MAS) theories and methods to participate in the exchange of necessary information among individuals. Each distributed generation unit participating in the economic dispatch performs data calculations according to the set distributed strategy, makes independent decisions, and adjusts its own output, collaboratively achieving the goal of economic dispatch. Currently, the main distributed algorithms applied in the field of power system economic dispatch include the distributed gradient descent algorithm [5], alternating direction method of multipliers (ADMM) [6,7], and consensus algorithm. Reference [5] proposes a distributed multi-step gradient descent method based on the upper bound of the second derivative to address the economic dispatch problem in microgrids. The distributed gradient algorithm is simple in principle and easy to implement. However, due to its poor performance in terms of convergence and robustness, it is more suitable for small-scale power systems. Reference [6] introduces a distributed economic dispatch algorithm based on the alternating direction method of multipliers (ADMM). This algorithm can handle both equality and inequality constraints simultaneously. ADMM exhibits good convergence and robustness, and it can also demonstrate excellent control performance in some non-convex problems. However, ADMM is sensitive to the selection of key parameters, and improper parameter selection may affect the algorithm’s convergence speed and stability. Additionally, due to the inherently decentralized nature of ADMM, it has not been widely adopted in economic dispatch.

The consensus algorithm is characterized by good convergence, strong robustness, and high flexibility, making it a pivotal method in the field of distributed control in power systems. It is widely applied in areas such as voltage/frequency control, power optimization control, harmonic mitigation, and economic dispatch [8,9,10,11]. Reference [12] adopted the consensus protocol and proved that the economic dispatch optimum was obtained when the incremental costs of all power generation were the same. References [13,14] adopted the leader–follower model based on the consensus algorithm to design the distributed economic dispatch system, in which the “leader” node acquires the data of system power differences, and the rest of the nodes communicate with each other to exchange the consensus variables, calculating and updating their outputs independently. As a counterpart, reference [15] investigated a distributed consistency improvement algorithm without a leader, which had faster convergence and higher stability.

With the increasing integration of high-proportion distributed energy resources and the surge in regulation demands, the deployment of various intelligent sensing terminals and high-speed communication links has significantly increased. Due to the deep coupling of information and physical systems in the power grid, when the information layer is compromised and malfunctions, the fault issues can propagate across domains to the physical layer, leading to equipment failures or power outages. Moreover, under a decentralized control architecture, attackers can target any communication node or link within the network, posing severe challenges to the defense against cybersecurity threats in power systems [16,17]. At present, information security issues have received sufficient attention in multiagent distributed control. The design of feasible distributed security resilience algorithms can effectively counteract cyber attacks and eliminate their associated adverse impacts. Reference [18] proposed a real-time distributed economic dispatch scheme for grid-connected microgrids to counteract cyber attacks. It established a detection and defense mechanism based on the reputation values of communication nodes, where the reputation values and communication weights of nodes decrease with the frequency of attacks. Once the reputation value falls below a predefined threshold, the node is isolated from the communication network, thereby mitigating the impact of the attacks. Reference [19] proposed a distributed resilience algorithm by integrating an event-triggered mechanism with a reputation protocol. The algorithm employs dynamic threshold detection and a node isolation strategy based on reputation value adjustments to counteract false data injection attacks. This approach effectively eliminates the incremental cost deviations caused by attacks, enabling the economic dispatch of integrated electricity and heat energy systems. These methods primarily focus on attack detection and isolation. While such approaches can mitigate the impact of cyber attacks, they may introduce control deviations due to the lack of communication information. Additionally, isolating the compromised distributed generation units and relying on the remaining distributed generation units for economic dispatch may not necessarily yield the most economical results.

Reference [20] proposed a resilient distributed algorithm based on trusted nodes, which achieved consensus among normal and trusted nodes after excluding selfish nodes. This idea of completely removing malicious information was then further expanded into a family of algorithms known as mean-subsequence-reduced (MSR) algorithms [21]. The idea of this class of methods was that normal nodes ranked the information values received from all of their neighbors in order of magnitude, and then removed the largest and smallest *F* (*F* representing the estimated upper limit of maliciously behaving nodes) values, and used only the remaining information to update their own state. The method may delete some normal information, thereby resulting in slower system convergence. To overcome this issue, weighted-MSR (W-MSR) [22] and event-MSR (E-MSR) [23] algorithms had been successively proposed. The former calculated the weighted average of the filtered valid information, and the latter achieved resilient consensus by reducing the frequency of information exchange between nodes through event-triggered mechanisms.

The local communication network topology is a determinant for the performance of the distributed economic dispatch system. It is currently common to use the connectivity index of the topology graph to study its influence on the convergence and robustness of the system. Reference [24] studied the convergence problem of distributed consistent economic dispatch under strong connected topology and proved that the strong connected communication network topology was a sufficient condition for the convergence of the economic dispatch algorithm. It was also shown in [25,26] that the communication topology could affect the convergence speed of the distributed consistent economic dispatch strategy. Reference [27] has shown that communication network topology will determine the ability of the distributed algorithms to counter the attacks carried out by the nodes. However, the MSR family of algorithms all need to remove information, which is equivalent to the switching of the network topology in each iteration. The traditional method of measuring network convergence with network connectivity has been challenged greatly. Providing the global network information needed to calculate connectivity in distributed mode is difficult. Reference [28] proved that when *F* nodes were attacked in a distributed system, at least 2*F* + 1 network connections must be provided to ensure security consensus. However, normal nodes are required to have at least some non-local information. Reference [29] further showed that the use of network connectivity in analyzing the convergence of the W-MSR algorithm under the directed graph was inappropriate because even if the connectivity of the directed graph was large, it was not enough to ensure the convergence of normal nodes. Reference [22] emphasized that a graph cannot achieve consensus even if its connectivity is large because the number of neighbor nodes in the complementary subset is insufficient, thereby causing each node to delete all useful information from outside its subset, so that it cannot reach consensus. Furthermore, this research further proposed a new topological property, namely network robustness, to describe the security consensus based on the M-MSR algorithm that uses only local information.

Through the analysis of existing research, research gaps can be summarized as follows:

(1) Attack detection and isolation methods are widely adopted in the research on information security for distributed economic dispatch. Although these methods are effective to some extent, the isolation of nodes may lead to the infringement of individual rights or impair the system’s power generation capability. Therefore, it is necessary to develop an algorithm that can resist attacks without isolating nodes, ensuring both the information security and system stability of distributed economic dispatch.
(2) In distributed systems, traditional methods typically rely on network connectivity to measure system convergence. However, in scenarios where network topology dynamically switches, the implementation of such methods faces significant challenges. Additionally, in distributed modes, it is difficult to provide the global network information required for calculating connectivity. Therefore, it is essential to explore new analytical methods to more effectively analyze the communication network topology of distributed systems.

Therefore, to overcome these challenges, this paper studies a secure distributed economic dispatch strategy aiming at strengthening the security of the system in the presence of information attacks. The major contributions of this paper are as follows: (1) A distributed secure economic dispatch method is formulated based on the W-MSR algorithm, which, to our best knowledge, is the first strategy that can ensure the security of a distributed economic dispatching system faced with information attacks. (2) The conditions that the proposed dispatch system under the W-MSR algorithm needs to meet to achieve a consistent communication network topology are given and proved on the basis of the robustness index.

The remainder of this paper is structured as follows: The attack modes and their impacts on a distributed dispatch system are introduced in Section 2. Section 3 proposes the secure dispatch strategy based on the W-MSR algorithm and Section 4 analyses the communication network based on a robust graph. The simulation results of proposed secure dispatch algorithm are provided in Section 5. Finally, in Section 6, the main findings of the paper are summarized.

## 2. Attack Modes and Their Impacts on a Distributed Dispatch System

### 2.1. Structure of the Distributed Economic Dispatch System

**Figure 1:**
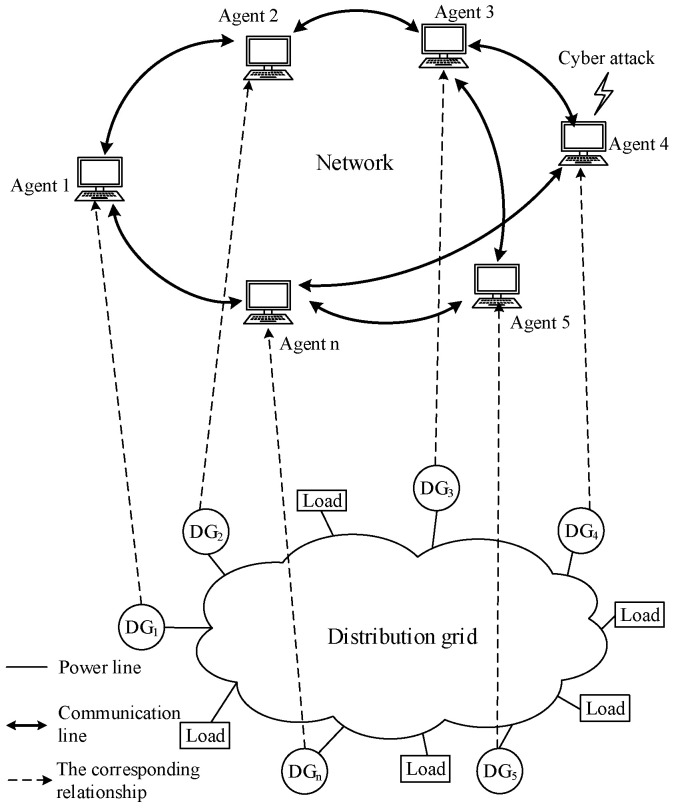
Structure of a distributed economic dispatch system under the concept of an MAS.

The distributed economic dispatch system is usually designed and implemented with the help of the MAS concept. As shown in Figure 1, each power generation unit that participates in economic dispatch corresponds to an agent with communication and autonomous computing capabilities, and each agent updates certain variable(s) (i.e., the consensus variable, which is usually the incremental cost of a power generation unit) of itself through an agreed protocol by interacting with information from its neighboring agents, and calculates its own output power. When the consensus variables of all agents take the same value, the system operates at its economic optimum.

In an MAS, the communication network equipped between agents (individuals) is abstracted by its topology graph, and graph theory is always applied to research the relevant characteristics of an MAS [30]. Treating individuals as the vertices and inter-individual communication links as the edges, the topology of a communication network with *n* agents can be represented as a graph ***G*** = (***V***, ***E***), where ***V*** = {1, 2, …, *n*} is the set of vertices, and *E*⊆***V*** × ***V*** is the set of edges. *N_i_* = {*j*∈***V***: (*i*,*j*)∈***E***} indicates the set of vertices in which each one has a direct communication link with vertex *i*, and *d_i_* = |*N_i_*| is the in-degree of vertex *i*. Define *J_i_* = *N_i_*∪{*i*}. *A*\*B* indicates a set that belongs to *A* but not *B*, and Z_≥*q*_ is a set of integers greater than or equal to *q*.

### 2.2. Node Attack Models

Compared with the centralized structure, a distributed system may suffer a larger range of information attacks caused by the decentralized decision-making fashion; that is, each agent can be a potential target of the attack. In distributed mode, even if only one agent may be attacked, it may destroy the global stability of the system. The model of an attack on the individuals/nodes of a distributed network usually includes two aspects, namely, the mode of operation and the scope of deployment. According to different modes of action, node attacks can be usually divided into several types, such as Crashed, Malicious, and Byzantine [31].

Representing the normal node set and the set of nodes under attack by ***V****_n_* and ***V****_m_* respectively, where $V_{n}\cup V_{m}=V$ and $V_{n}\cap V_{m}=Ø$, various attack models are defined as follows:

**Definition** **1**  (Crashed attack)**.** *For a node i∈****V****_m_, if it stops to update its state at a certain time, and the information that has not been updated is transmitted to its neighbor nodes, then this behavior is called a Crashed attack, and node i is a crashed node.*

The attack model is as follows:

(1) $$x_{i}^{j}[k]=x_{i}[k_{0}],k\in [k_{0},k_{\mathrm{end}}]$$

where *j* is a neighbor node of *i*; $x_{i}^{j}[k]$ denotes that node *j* receives information from node *i* at the iteration cycle *k*; $k_{0}$ is the moment when the attack starts; and $k_{\mathrm{end}}$ is the moment when the attack ends.

**Definition** **2**  (Malicious attack)**.** *For a node i∈****V****_m_, if it does not update its state according to the agreed protocol at some moments, but the information passed to the neighbor nodes at any moment is consistent, then this behavior is called a Malicious attack, and the individual i is a malicious node.*

The attack model is as follows:

(2) $$x_{i}^{j}[k]=\omega_{j}x_{i}[k],\;k\in [k_{0},k_{\mathrm{end}}]$$

where $\omega_{j}$ denotes the scaling rate of the transmitted information and all neighbor nodes of *i* have the same scaling rate.

**Definition** **3**  (Byzantine attack)**.** *For a node i∈****V****_m_, if it does not update its state according to the agreed protocol at some moments or the information passed to the different neighbor nodes at some moments is inconsistent, then the behavior is called a Byzantine attack, and the node i is a Byzantine node.*

The attack model is as follows:

(3) $$x_{i}^{j}[k]=\omega_{j}x_{i}[k],\;k\in [k_{0},k_{\mathrm{end}}]$$

where all neighbor nodes of *i* have different scaling rates.

In addition to the mode of action, the scope of deployment of a node attack is also important. Generally, the deployment range can be expressed by a constant. Currently, three models are commonly used: the *F*-total (*F*-total deployment) model, *F*-local (*F*-local deployment) model, and *f*-fraction local (*f*-local proportion deployment) model. These are defined as follows:

**Definition** **4**  (F-total model)**.** *A maximum of F nodes have attack behavior in the n node system, that is, |**V**_m_| ≤ F, F∈**Z**_+_. |·| represents the number of elements in the set, and **Z**_+_ represents a set of positive integers.*

**Definition** **5**  (F-local model)**.** *A normal node has a maximum of F neighbor nodes that have attack behavior in the n node system, that is, |**V**_m_∩N_i_| ≤ F, $\forall i\in V_{n}$* *, F* *∈* ***Z*** *_+_.*

**Definition** **6**  (f-fraction local model)**.** *In an n node system, the proportion of attacking nodes in the neighbors of a normal node does not exceed f, that is, |**V**_m_∩N_i_| ≤ f |N_i_|, $\forall i\in V_{n}$* *, 0 ≤ f ≤ 1.*

### 2.3. Influences of the Attacks

The traditional consensus-based distributed economic scheduling completely relies on the information from neighboring nodes, and all nodes update the consensus variable using the same protocol. In a Crashed attack, the attacked node has always passed the wrong information to its neighbor nodes. The results calculated by other normal nodes according to the consensus protocol are inconsistent with the results of normal nodes that cannot receive error information, thereby leading to the failure of the dispatch task. More seriously, the system power balance may be disrupted because of the presence of erroneous information. For Malicious or Byzantine attacks, short-term attacks cause temporary damage to the system. As the attack stops, the system can re-converge to the optimum. A long attack will have the same effect as a Crashed attack. The traditional distributed consensus algorithm cannot achieve the dispatch goal after being attacked, and because the distributed system has a highly decentralized computing decision-making fashion, a single node attack is likely to affect the stability of the entire system. Therefore, the safe distributed economic dispatch strategy in the presence of attacks should be deeply investigated.

## 3. Secure Dispatch Strategy Based on W-MSR Algorithm

### 3.1. Consensus Algorithm

At present, distributed economic dispatch strategies are mostly based on the consensus protocols. The first-order linear discrete consensus protocol commonly used by *n* agent systems can be expressed as follows:

(4) $$x_{i}[k+1]=\sum_{j\in J_{j}[k]}w_{ij}[k]x_{j}[k]$$

where *x_i_*[*k*] represents the agent value that corresponds to node *i* at the iteration cycle *k*; and *w_ij_*[*k*] represents the weight of information exchanged between nodes *i* and *j* at cycle *k*, which usually needs to satisfy the following:

(5) $$\left\{\begin{array}{l} w_{ij}[k]=0,\;j\notin J_{i}[k]\\ w_{ij}[k]\geq \alpha ,\;j\in J_{i}[k]\\ \sum_{j=1}^{n}w_{ij}[k]=1 \end{array}\right.$$

where α is a constant between (0, 1).

On the premise that *w_ij_*[*k*] satisfies Formula (5), when the time-invariant communication network topology of the system has a spanning tree, each agent can update its state iteratively according to protocol (1), and the state of all agents eventually reaches the same, which is as follows:

(6) $$x_{1}=x_{2}=\cdots =x_{n}$$

and vice versa [32].

Weight *w_ij_*[*k*] largely determines the convergence speed for a consensus-based distributed algorithm. It has been shown in [31] that when the topology is a fixed undirected graph, the weight optimization problem can be converted into a semidefinite program (SDP) problem to obtain a faster convergence speed. However, solving the SDP problem requires global information, and weight optimization does not have a significant impact on the convergence speed when the network scale is relatively small. Therefore, this paper uses the following weighting scheme that only requires local information.

(7) $$w_{ij}[k]=\frac{1}{1+d_{i}(k)},j\in J_{i}[k]$$

### 3.2. W-MSR Algorithm

The traditional distributed economic dispatch system can achieve the consensus result according to the linear update rule given by Formula (4) and the weight value given by Formula (7). However, information security in a distributed network requires special attention since it cannot defend against attacks.

According to the definition of the attack model, the attacked node propagates wrong or even harmful information to the neighbor nodes. The normal nodes need to update their local states safely by accurately removing the information from the nodes with malicious behaviors, which is the main idea of the W-MSR algorithm. Taking the *F*-local model as an example, the node *i* obtains information about itself and all neighbor nodes at iteration cycle *k*. Among them, at most, *F* neighbor nodes may be attacked or malicious, and the attacked information evidently deviates from the consistent value. Thus, node *i* should remove the received values that are strictly greater than and less than its own value, and update its own state with the remaining correct information. The specific implementation of the W-MSR algorithm is presented as follows:

1. Node *i* obtains the data for itself and all of its neighbors at cycle *k* and arranges them by size.
2. If there are less than *F* neighbors strictly greater than its own value *x_i_*[*k*], then all values strictly greater than itself must be removed. Otherwise, the first *F* maximum values shall be removed. Similarly, if there are less than *F* neighbors strictly less than its own value *x_i_*[*k*], then all values strictly less than itself must be removed. Otherwise, *F* minimums must be ignored.
3. Let *R_i_*[*k*] represent the set of neighbors removed by node *i* in Step 2, and the state update role of node *i* is modified to the following:

(8) $$x_{i}[k+1]=\sum_{j\in J_{j}[k]\backslash R_{f}[k]}w_{ij}[k]x_{j}[k]$$

where *J_i_*[*k*]/*R_i_*[*k*] represents the set after *J_i_*[*k*] after removing *R_i_*[*k*]. The weight *w_ij_*[*k*] still should satisfy Formula (5); one of the simple ways to adjust *w_ij_*[*k*] is for any *j*∈*J_i_*[*k*]\*R_i_*[*k*], *w_ij_*[*k*] = 1/(1 + *d_i_*[*k*] − |*R_i_*[*k*]|).

**Figure 2:**
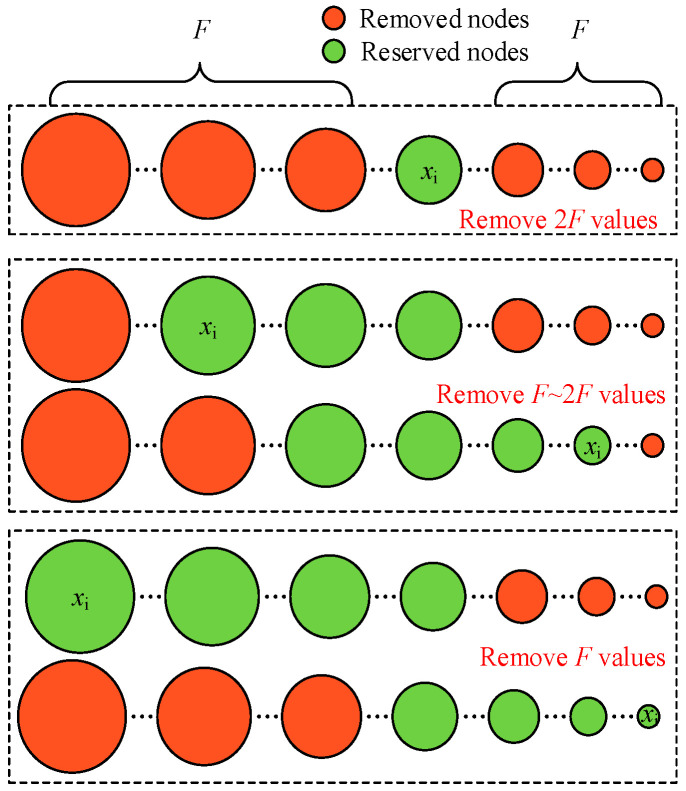
Principle of the node information filtering.

Step 2 suggests that the number of removed values is related to the value of node *i* in cycle *k*, and the MSR algorithm removes the first *F* maximum values and the first *F* minimum values in each iteration cycle. Figure 2 conceptually illustrates the principle of the value filtering of the W-MSR algorithm in coping with an *F*-local attack.

### 3.3. Distributed Secure and Economic Dispatch Strategy

The goal of distributed economic dispatch is to minimize the total power generation cost under the premise of meeting the condition constraints. Thus, the objective function is usually defined as follows:

(9) $$\min F=\sum_{i=1}^{n}F_{i}\left(P_{Gi}\right)$$

where *n* is the number of power generation units in the system, and *P_Gi_* and *F_i_*(*P_Gi_*) are the output power and cost function of the power-generating unit *i*, respectively. Although differences in the power generation cost functions of different types of power-generating units are observed, most are currently given in the form of a quadratic convex function [33], which is as follows:

(10) $$F_{i}\left(P_{Gi}\right)=a_{i}P_{Gi}^{2}+b_{i}P_{Gi}+c_{i}$$

where *a_i_*, *b_i_*, and *c_i_* are the coefficients of the second, first, and constant terms of the cost function for power generation unit *i*, respectively.

Power balance is a condition that needs to be satisfied for power system operation. Thus, the equation constraint of the economic dispatch optimization model is as follows:

(11) $$\sum_{i=1}^{n}P_{Gi}=P_{D}$$

where *P_D_* is the total load of the system.

The inequality constraint of the optimization model is as follows:

(12) $$P_{Gi}^{\min}\leq P_{Gi}\leq P_{Gi}^{\max}$$

where $P_{Gi}^{\min}$ and $P_{Gi}^{\max}$ are the maximum and minimum values of the output power-generating unit *i*, respectively.

Using the Lagrangian multiplier, the optimization model can be expressed as follows:

(13) $$\left\{\begin{array}{l} \min\;F=\sum_{i=1}^{n}F_{i}(P_{Gi})+\lambda \Delta P\\ s.t.\;P_{Gi}^{\min}\leq P_{Gi}\leq P_{Gi}^{\max} \end{array}\right.$$

where *λ* is a Lagrangian multiplier, which is the incremental cost of each power-generating unit, and $\Delta P=P_{D}-\sum_{i=1}^{n}P_{Gi}$ is the system power deficit.

The partial derivative of *λ* and P_Gi_ should be determined and set to zero; hence, the following:

(14) $$\left\{\begin{array}{l} \frac{\partial F}{\partial P_{Gi}}=\frac{dF_{i}\left(P_{Gi}\right)}{dP_{Gi}}-\lambda \left(1-\frac{\partial P_{D}}{\partial P_{Gi}}\right)=0\\ \frac{\partial F}{\partial \lambda }=P_{D}-\sum_{i=1}^{n}P_{Gi}=0 \end{array}\right.$$

The simultaneous solution of the aforementioned equations shows that the optimal solution for economic dispatch is achieved when all power generation units have the same value of λ, since the cost function is a convex one. Therefore, economic dispatch optimization can be transformed into a consensus problem taking *λ_i_* as the consensus variable. Considering that the distributed economic dispatch system may be attacked, the W-MSR algorithm is applied to obtain the consensus variable update rule:

(15) $$\lambda_{i}[k+1]=\sum_{j\in J_{i}[k]}w_{ij}[k]\lambda_{j}[k]+\varepsilon \Delta P$$

where *ε* is the convergence coefficient, a positive number, and the system does not converge if it is too large [34].

Each power-generating unit determines the contribution based on the incremental cost update value obtained by Formula (14):

(16) $$P_{Gi}[k]=\frac{\lambda_{i}[k]-b_{i}}{a_{i}}$$

Considering the power constraints, Formula (14) can be changed into the following:

(17) $$P_{Gi}[k]=\left\{\begin{array}{ll} P_{Gi}^{\min} & \frac{\lambda_{i}[k]-b_{i}}{a_{i}}\leq P_{Gi}^{\min}\\ \frac{\lambda_{i}[k]-b_{i}}{a_{i}} & P_{Gi}^{\min}\leq \frac{\lambda_{i}[k]-b_{i}}{a_{i}}\leq P_{Gi}^{\max}\\ P_{Gi}^{\max} & \frac{\lambda_{i}[k]-b_{i}}{a_{i}}\geq P_{Gi}^{\max} \end{array}\right.$$

Through these steps, the safe economic dispatch in distributed mode can be obtained while maintaining the consensus of the optimal solution with the centralized method:

(18) $$\left\{\begin{array}{l} \lambda_{i}^{*}=\left(P_{D}+\sum_{i=1}^{n}\frac{b_{i}}{2a_{i}}\right)/\sum_{i=1}^{n}\frac{1}{2a_{i}}\\ P_{Gi}^{*}=\left(\lambda_{i}^{*}-b_{i}\right)/\left(2a_{i}\right) \end{array}\right.$$

**Figure 3:**
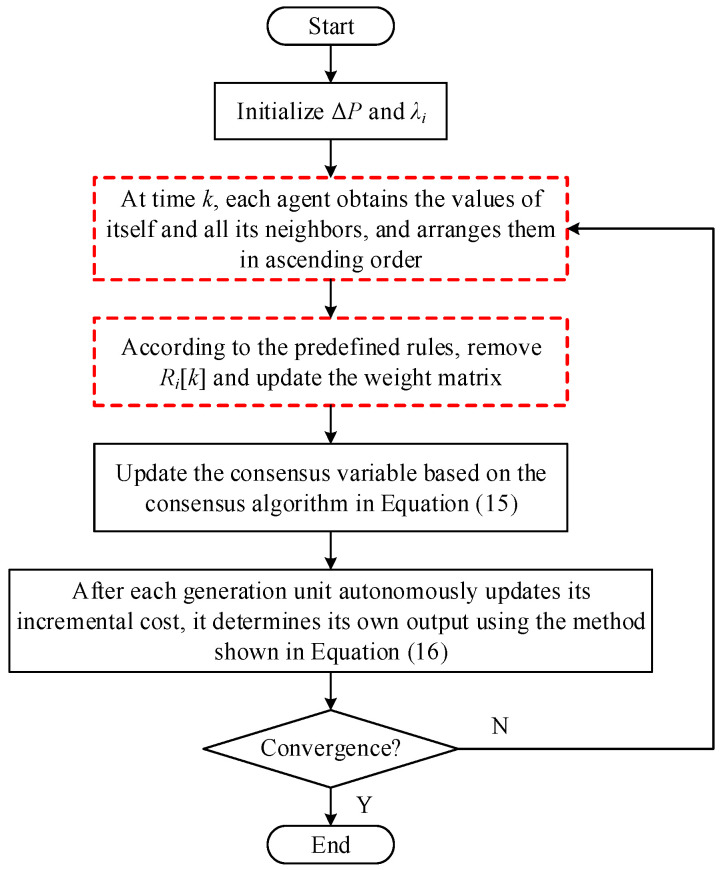
Flow chart of distributed security consensus economic dispatch to deal with attacks.

In summary, the secure W-MSR-based distributed economic dispatch process to deal with attack behavior is depicted in Figure 3.

Compared with traditional distributed economic dispatching, the distributed safe economic dispatching strategy in this paper adopts the W-MSR algorithm to improve the updating rules of consensus variables. The individual ensures that only the correct information is used to update the consensus variables by filtering out the wrong or harmful information to obtain the optimal solution for economic dispatch. However, this process will involve the removal of information and the change in weights; that is, even if the communication network topology is fixed, it will also “induce” the dynamic topology. Therefore, the conditions that the communication network topology of the economic dispatch system based on the W-MSR algorithm needs to meet should be investigated.

## 4. Topology Analysis of Communication Network Based on a Robust Graph

This study analyzes the communication network topology of the economic dispatch system based on the W-MSR algorithm according to the network robustness index. The following definitions of available sets are given:

**Definition** **7**  (r-available set)**.** *For a graph G = (V, E) and a nonempty subset S*⊂*V, if there are at least r neighbors of node i∈S from outside the set S, that is, |N_i_\S| ≥ r, r∈Z_≥0_, then the set S is called an r-available set.*

**Definition** **8**  ((r, s)-available set)**.** *For a graph G = (V, E) and a nonempty node subset S*⊂*V, if there are no less than s nodes in set S such that each one has at least r neighbor nodes from outside the set S, let set X^r^_S_ = {i∈S: |N_i_\S| ≥ r}, meeting |X^r^_S_| ≥ s, r, s∈Z_≥0_. Then, the set S is called an (r, s)-available set. The (r, 1)-available set is naturally an r-available set.*

**Figure 4:**
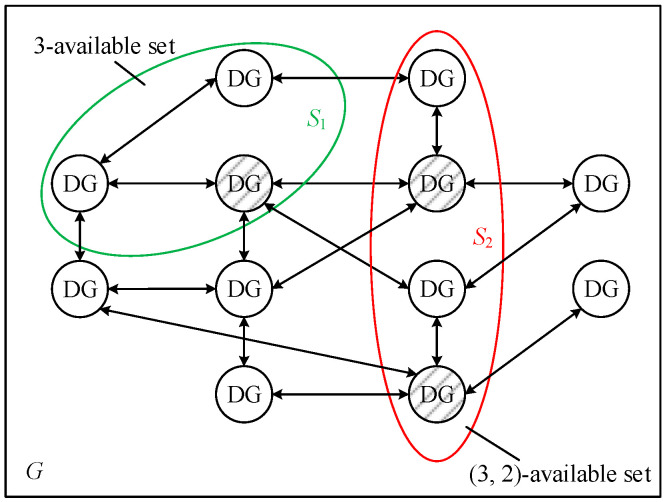
Illustration of the (r, s)-available set.

Take the communication network topology *G* shown in Figure 4 as an example to illustrate the aforementioned concept. *S*_1_ and *S*_2_ are the divided subsets of *G* (disjoint subset). Let *r* = 3. One power generation unit (represented by a shadow filled cycle) in *S*_1_ can receive the information from at least three power generation units in the off-set of *S*_1_; hence, *S*_1_ is a three-available set. Similarly, two power generation units in *S*_2_ can receive the information from at least three power generation units in the off-set of *S*_2_; hence, *S*_2_ is a (3, 2)-available set.

Based on the available set concept, the robust graph is further defined as follows:

**Definition** **9** (r-robust graph [30])**.** *A graph G = (V, E) is an r-robust graph if, for any pair of divided subsets S_1_ and S_2_ of V, at least one is an r-available set.*

**Definition** **10** ((r, s)-robust graph [22])**.** *For a graph G = (V, E) (the number of nodes n ≥ 2), and S_1_ and S_2_ as any pair of divided subsets of V, G is an (r, s)-robust graph if the following condition is satisfied:*

(19) $$\left|X_{S_{1}}^{F+1}\right|+\left|X_{S_{2}}^{F+1}\right|\leq F$$

The conditions to be satisfied by the local communication network topology for implementing a distributed safe and economic dispatch strategy based on the W-MSR algorithm are given in the following theorem.

**Theorem** **1.** *For the economic dispatch system with n distributed power units (Figure 1), the communication topology is G = (V, E), and each normal node updates the consensus variable value according to Formula (15). The W-MSR algorithm can reach the optimal solution of economic dispatch if and only if G is a (F + 1, F + 1)-robust graph.*

**Proof of Theorem** **1.** (Necessity.) If *G* is not an (*F* + 1, *F* + 1)-robust graph, then the nonempty disjoint subsets *S*_1_ and *S*_2_ do not satisfy any condition of Formula (19). Assume that the initial states of each node in *S*_1,_ *S*_2_ are *a* and *b*, respectively, and *a* < *b*, and set the initial state of others in the range of (*a*, *b*). Given that $\left|X_{S_{1}}^{F+1}\right|+\left|X_{S_{2}}^{F+1}\right|\leq F$, it can be supposed that the nodes in $X_{S_{1}}^{F+1}$ and $X_{S_{2}}^{F+1}$ are all of the attacked nodes and remain to transmit their initial values. Considering $\left|X_{S_{1}}^{F+1}\right|<\left|S_{1}\right|$ and $\left|X_{S_{2}}^{F+1}\right|<\left|S_{2}\right|$, at least one normal node can be found in *S*_1_ and *S*_2_. The normal node(s) cannot reach an agreement because it(they) can only remove *F* or less than *F* values received from the neighbor nodes.

(Sufficiency.) Define the set of all normal nodes by N, and *N = |N|. M*[*t*] and *m*[*t*] are defined as the upper and lower values of the normal node at time *t*, respectively. *M*[*t*] and *m*[*t*] are the monotone bounded functions of *t* and their limits are defined as *A_M_* and *A_m_*. If *A_M_* = *A_m_*, the normal nodes can reach consensus. Next, this situation will be proven by contradiction.

Assume *A_M_* > *A_m_*. Let constant $\epsilon_{0}$ > 0, and make $A_{M}-\epsilon_{0}>A_{m}+\epsilon_{0}$. At moment *t* for any positive real value $x_{i}[t]>A_{M}-\epsilon_{i}$, let *X_M_*(*t*, $\epsilon_{i}$) = { *i*∈*V*:$x_{i}[t]>A_{M}-\epsilon_{i}$}, which corresponds to all of the nodes greater than $A_{M}-\epsilon_{i}$; let *X_m_*(*t*, $\epsilon_{i}$) = { *i*∈*V*:$x_{i}[t]<A_{m}+\epsilon_{i}$}, which refers to all of the nodes smaller than $A_{m}+\epsilon_{i}$. The definition of $\epsilon_{0}$ shows that *X_M_*(*t*, $\epsilon_{0}$) and *X_m_*(*t*, $\epsilon_{i}$) are disjoint.

Fix $\epsilon <\frac{\alpha^{N}}{1-\alpha^{N}}\epsilon_{0}$ (*α* is the line weight), which meets $\epsilon_{0}>\epsilon >0$. Set $t_{\epsilon }$ to $M[t]<A_{M}+\epsilon$ and $m[t]>A_{m}-\epsilon$, $\forall t\geq t_{\epsilon }$ ($t_{\epsilon }$ exists according to the definition of convergence). Nonempty sets *X_M_*(*t*, $\epsilon_{0}$) and *X_m_*(*t*, $\epsilon_{0}$) are also disjoint. Because *G* is an (*F* + 1, *F* + 1)-robust graph, the number of malicious nodes is not greater than *F*. Thus, a normal node with at least *F* + 1 neighbors outside its set exists. Without losing generality, assume that normal node $i\in X_{M}(t_{\epsilon },\epsilon_{0})\cap N$ has at least *F* + 1 neighbors outside the set $X_{M}(t_{\epsilon },\epsilon_{0})$. According to the definition, the maximum value of these neighbor nodes is $A_{M}-\epsilon_{0}$, and at least one of these values is used by node *i*. Notably, at each step, the value of each normal node is a convex combination of its own value, and the value it uses from its neighbors and each coefficient in the combination are bounded by *α*. At moment $t_{\epsilon }$, the maximum value used by node *i* is $M[t_{\epsilon }]$. If setting $M[t_{\epsilon }]$ has the largest possible weight, then the following is obtained:

(20) $$\begin{array}{cc} x_{i}\left[t_{\epsilon }+1\right] & \leq (1-\alpha )M\left[t_{\epsilon }\right]+\alpha \left(A_{M}-\epsilon_{0}\right)\\ & \leq (1-\alpha )\left(A_{M}+\epsilon \right)+\alpha \left(A_{M}-\epsilon_{0}\right)\\ & \leq A_{M}-\alpha \epsilon_{0}+(1-\alpha )\epsilon \end{array}$$

The limit is also suitable for normal nodes that are out of the set $X_{M}(t_{\epsilon },\epsilon_{0})$, because these nodes update only using their own value. Similarly, if node $j\in X_{m}(t_{\epsilon },\epsilon_{0})\cap N$ has *F* + 1 neighbors outside set $X_{m}(t_{\epsilon },\epsilon_{0})$, then $x_{j}\left[t_{\epsilon }+1\right]\geq A_{m}+\alpha \epsilon_{0}-(1-\alpha )\epsilon$, and normal nodes that are out of the set $X_{m}(t_{\epsilon },\epsilon_{0})$ have this limit.

Define $\epsilon_{1}=\alpha \epsilon_{0}-(1-\alpha )\epsilon$, which meets $0<\epsilon <\epsilon_{1}<\epsilon_{0}$. Take into account $X_{M}(t_{\epsilon }+1,\epsilon_{1})$ and $X_{m}(t_{\epsilon }+1,\epsilon_{1})$. Because at least one normal node in set $X_{M}(t_{\epsilon },\epsilon_{0})$ reduces to $A_{M}-\epsilon_{1}$ (or smaller) or at least one normal node in set $X_{m}(t_{\epsilon },\epsilon_{0})$ increases to $A_{m}+\epsilon_{1}$ (or larger), it must be that either $\left|X_{M}(t_{\epsilon }+1,\epsilon_{1})\cap N\right|<\left|X_{M}(t_{\epsilon },\epsilon_{0})\cap N\right|$ or $\left|X_{m}(t_{\epsilon }+1,\epsilon_{1})\cap N\right|<\left|X_{m}(t_{\epsilon },\epsilon_{0})\cap N\right|$, or both. Set $X_{M}(t_{\epsilon }+1,\epsilon_{1})$ and $X_{m}(t_{\epsilon }+1,\epsilon_{1})$ are also disjoint because of $\epsilon_{1}<\epsilon_{0}$. For *j* ≥ 1, define $\epsilon_{j}=\alpha \epsilon_{j-1}-(1-\alpha )\epsilon$, and $\epsilon_{j}<\epsilon_{j-1}$. If both sets $X_{M}(t_{\epsilon }+1,\epsilon_{j})$ and $X_{m}(t_{\epsilon }+1,\epsilon_{j})$ have normal nodes, the above analysis can be repeated at time $t_{\epsilon }+j$. In addition, at time $t_{\epsilon }+j$, either $\left|X_{M}(t_{\epsilon }+j,\epsilon_{j})\cap N\right|<\left|X_{M}(t_{\epsilon }+j-1,\epsilon_{j-1})\cap N\right|$ or $\left|X_{m}(t_{\epsilon }+j,\epsilon_{j})\cap N\right|<\left|X_{m}(t_{\epsilon }+j-1,\epsilon_{j-1})\cap N\right|$, or both. There must exist a moment $t_{\epsilon }+T$ (*T ≤ N*) where both $X_{M}(t_{\epsilon }+T,\epsilon_{T})\cap N$ and $X_{m}(t_{\epsilon }+T,\epsilon_{T})\cap N$ are empty because $\left|X_{M}(t_{\epsilon }+1,\epsilon_{0})\cap N\right|+\left|X_{m}(t_{\epsilon },\epsilon_{0})\cap N\right|\leq N$. In the former scenario, the value of each normal node at moment $t_{\epsilon }+T$ is mostly $A_{M}-\epsilon_{T}$. In the latter scenario, the value of each normal node at moment $t_{\epsilon }+T$ is no less than $A_{m}+\epsilon_{T}$.

The following explanation will prove that $\epsilon_{T}>0$ conflicts with the fact that the maximum value converges monotonically to *A_M_* (the former scenario) or the minimum value converges monotonically to *A_m_* (the latter scenario). The reason is the following:

(21) $$\begin{array}{cc} \epsilon_{T} & =\alpha \epsilon_{T-1}-(1-\alpha )\epsilon \\ & =\alpha^{2}\epsilon_{T-2}-\alpha (1-\alpha )\epsilon -(1-\alpha )\epsilon \\ & \vdots \\ & =\alpha^{T}\epsilon_{0}-(1-\alpha )\left(1+\alpha +\cdots +\alpha^{T-1}\right)\epsilon \\ & =\alpha^{T}\epsilon_{0}-\left(1-\alpha^{T}\right)\epsilon \\ & \geq \alpha^{N}\epsilon_{0}-\left(1-\alpha^{N}\right)\epsilon \end{array}$$

Since $\epsilon <\frac{\alpha^{N}}{1-\alpha^{N}}\epsilon_{0}$, $\epsilon_{T}>0$, which contradicts the above analysis. Therefore, $\epsilon_{0}=0$ must obtain *A_M_* = *A_m_*. □

## 5. Simulation Verification

### 5.1. Configuration of the Dispatch System

The IEEE-39 bus distribution network was simulated and analyzed to prove the correctness and feasibility of the strategy presented in this paper [33]. We designed two case studies, connecting 10 and 20 distributed generation units to the network, respectively, to analyze the performance of the proposed algorithm as the scale of the communication network expands. Table 1 lists the basic parameters of each power generation unit.

**Figure 5:**
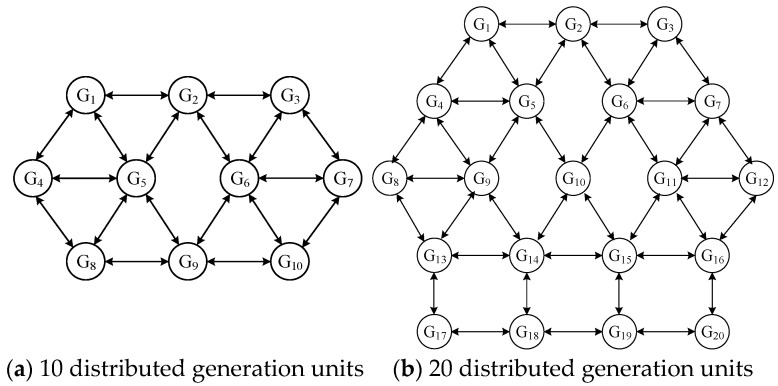
Topology of the communication network.

The initial power of each unit was set to the minimum value of the respective output power, and the initial load of the system was 3440 MW. Figure 5 shows the communication network topology among 10 distributed power generation units.

### 5.2. Analysis of the System with 10 Distributed Generation Units

#### 5.2.1. Simulation for Crashed Attack

**Figure 6:**
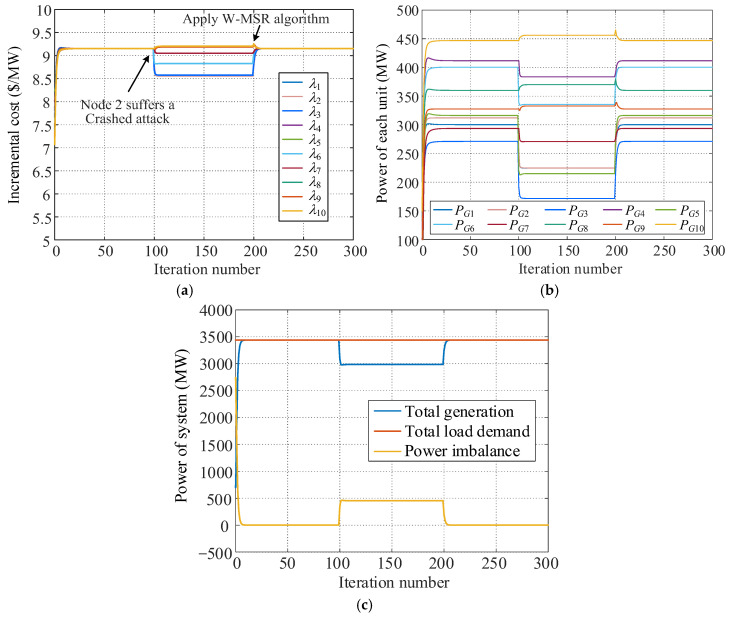
Stimulation results under Crashed attack. (**a**) Incremental cost of each unit. (**b**) Power of each unit. (**c**) Power of the system.

Each agent is assumed to adopt the traditional consensus-based distributed economic dispatch strategy (hereinafter referred to as the consensus algorithm) at the beginning of simulation, and no node attack occurs. When *k* = 100 (*k* is the iteration cycle), node 2 is under a Crashed attack, and the information sent to its neighbor nodes in the subsequent iteration process remains at its initial value *λ*_2_[0]. When *k* = 200, the proposed distributed secure economic dispatch strategy based on the W-MSR algorithm (hereinafter referred to as W-MSR algorithm) is activated. The obtained simulation results are shown in Figure 6, and the key information for each simulation stage is listed in Table 2.

Figure 6 and Table 2 show that for the consensus algorithm, the consensus variables of the ten power units begin to deviate from the same value of 9.152 USD/MW right after the Crashed attack and are different from each other. The power balance of the system is also destroyed, and the total generating power is less than the total load demand by 455 MW. Therefore, the distributed economic dispatch system using the traditional consensus algorithm cannot achieve economic dispatch goals after being attacked, and a single node attack may affect the stability of the entire system. After adopting the proposed W-MSR algorithm, the consensus variable of each unit rapidly re-converges to λ* = 9.1522 USD/MW, which is consistent with the calculation result of Formula (18). Moreover, the attacked nodes are not isolated, and the outputs of the generator units remain the same as when no node attacks occur. The overall power generation capability of the system is not compromised, and the dispatch system once again achieves power balance. The simulation results demonstrate that this strategy can effectively address collapse attacks, ensuring the safe and stable operation of the distributed dispatch system in the most economical state.

#### 5.2.2. Simulation for Byzantine Attack

**Figure 7:**
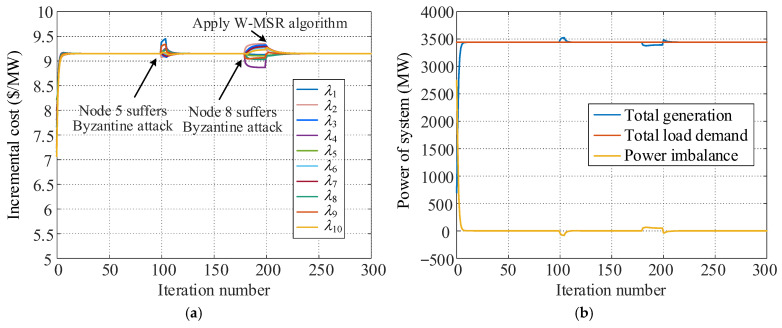
Simulation results under Byzantine attack. (**a**) Incremental cost of each unit. (**b**) Power of the system.

Node 5 is assumed to suffer a Byzantine attack from *k* = 100 to *k* = 105 and transmits inconsistent information to its different neighbor nodes. Among these neighbor nodes, 1.1*λ*_5_ is transmitted to node 1, 0.9*λ*_5_[*k*] is sent to node 2, 1.08*λ*_5_[*k*] is sent to node 9, and *λ*_5_[*k*] is sent to other neighbor nodes. At *k* = 180, node 8 is attacked by Byzantine continuously, and 0.92*λ*_8_[*k*] is transmitted to node 4, 0.95*λ*_8_[*k*] is sent to node 9, and *λ*_8_[*k*] is sent to other neighbor nodes. The proposed W-MSR algorithm is applied at *k* = 200. Figure 7 shows the variations in the consensus variables and system power in these scenarios.

Figure 7 reveals that when the traditional consensus algorithm is adopted, the two Byzantine attacks also prevent the consensus variables of the dispatch system from converging to the same value, and the system power balance is destroyed. However, the influence of this type of attack on the stability of the dispatch system is related to the duration of the attack. Specifically, when the attack time is short, such as the attack at node 5, the dispatch system can return to the optimal state and run stably after a certain number of iteration cycles automatically. A long-term Byzantine attack, such as the attack at node 8, may hinder the consensus variables from converging to the same value, thereby causing continuously unbalanced power in the system. As long the W-MSR algorithm is adopted, the economic dispatch system can resist continuous Byzantine attacks effectively and ensure that the system converges to a consistent state and runs stably at the optimal value.

### 5.3. Analysis of the System with 20 Distributed Generation Units

#### 5.3.1. Simulation for Crashed Attack

**Figure 8:**
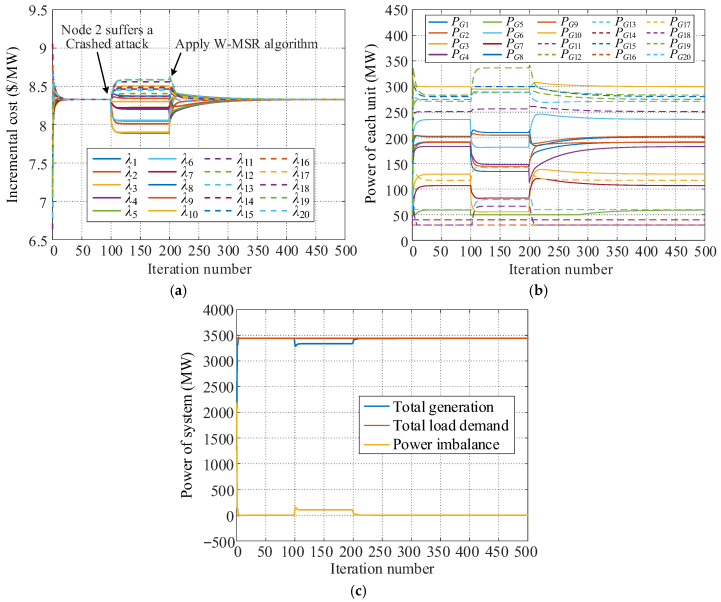
Stimulation results under Crashed attack. (**a**) Incremental cost of each unit. (**b**) Power of each unit. (**c**) Power of the system.

For the system with 20 distributed generation units, the simulation for Crashed attack is conducted with the specific attack scenarios consistent with those described in Section 5.2.1. At *k* = 100 (*k* is the iteration cycle), node 2 is under a Crashed attack. When *k* = 200, the proposed distributed secure economic dispatch strategy based on the W-MSR algorithm is activated. The obtained simulation results are shown in Figure 8, and the key information for each simulation stage is listed in Table 3.

Figure 8 and Table 3 show that for the consensus algorithm, the consensus variables of the twenty power units begin to deviate from the same value of 8.329 USD/MW right after the Crashed attack and are different from each other. The power balance of the system is also destroyed, and the total generating power is less than the total load demand by 103.55 MW. Therefore, the distributed economic dispatch system using the traditional consensus algorithm cannot achieve economic dispatch goals after being attacked, and a single node attack may affect the stability of the entire system. After adopting the proposed W-MSR algorithm, the consensus variable of each unit rapidly re-converges to λ* = 8.329 USD/MW. Moreover, the attacked nodes are not isolated, and the outputs of the generator units remain the same as when no node attacks occur. The overall power generation capability of the system is not compromised, and the dispatch system once again achieves power balance. The simulation results demonstrate that this strategy can effectively address collapse attacks, ensuring the safe and stable operation of the distributed dispatch system in the most economical state.

#### 5.3.2. Simulation for Byzantine Attack

**Figure 9:**
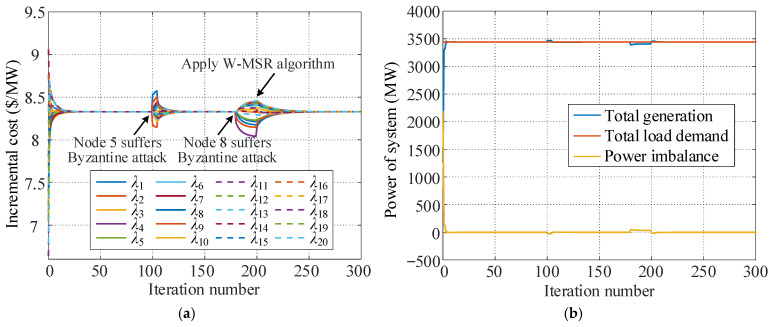
Simulation results under Byzantine attack. (**a**) Incremental cost of each unit. (**b**) Power of the system.

For the system with 20 distributed generation units, the simulation for Byzantine attack is conducted with the specific attack scenarios consistent with those described in Section 5.2.2. Node 5 is assumed to suffer a Byzantine attack from *k* = 100 to *k* = 105 and transmits inconsistent information to its different neighbor nodes. At *k* = 180, node 8 is attacked by Byzantine continuously. The proposed W-MSR algorithm is applied at *k* = 200. Figure 9 shows the variations in the consensus variables and system power in these scenarios.

Figure 9 reveals that when the traditional consensus algorithm is adopted, the two Byzantine attacks also prevent the consensus variables of the dispatch system from converging to the same value, and the system power balance is destroyed. However, the influence of this type of attack on the stability of the dispatch system is related to the duration of the attack. Specifically, when the attack time is short, such as the attack at node 5, the dispatch system can return to the optimal state and run stably after a certain number of iteration cycles automatically. A long-term Byzantine attack, such as the attack at node 8, may hinder the consensus variables from converging to the same value, thereby causing continuously unbalanced power in the system. As long as the W-MSR algorithm is adopted, the economic dispatch system can resist continuous Byzantine attacks effectively and ensure that the system converges to a consistent state and runs stably at the optimal value.

### 5.4. Comparative Analysis of Case Study Results

From the simulation results in Section 5.2 and Section 5.3, it can be observed that as more distributed generation units are integrated into the system and the communication network scales up, the proposed distributed secure economic dispatch strategy based on the W-MSR algorithm effectively withstands various modes of node attack, ensuring the secure and stable operation of the distributed dispatch system at the optimal solution. Moreover, the algorithm does not employ isolation measures, and the final result is the most economical dispatch achieved through the collective participation of all distributed generation units, thereby guaranteeing the economic efficiency of system operation. Table 4 presents the number of iterations required for the system to reconverge to the optimal solution using the W-MSR algorithm after being subjected to attacks in the two case study systems.

From Table 4, it can be observed that as the network scale expands, the time required for the W-MSR algorithm to counteract attacks slightly increases. Additionally, the comparison reveals that with the expansion of the network scale, the proposed distributed secure economic dispatch strategy based on the W-MSR algorithm requires more iterations to handle Crashed attacks, while the number of iterations needed to address Byzantine attacks remains almost unchanged. This is because a Crashed attack involves nodes ceasing state updates and propagating information that has not been updated to their neighboring nodes. As the network scale grows, this behavior of not updating states poses greater challenges to the convergence of the consensus protocol, requiring all agents to take longer to converge to a consistent value.

### 5.5. Verification of Sufficient and Necessary Conditions of Communication Network Topology

**Figure 10:**
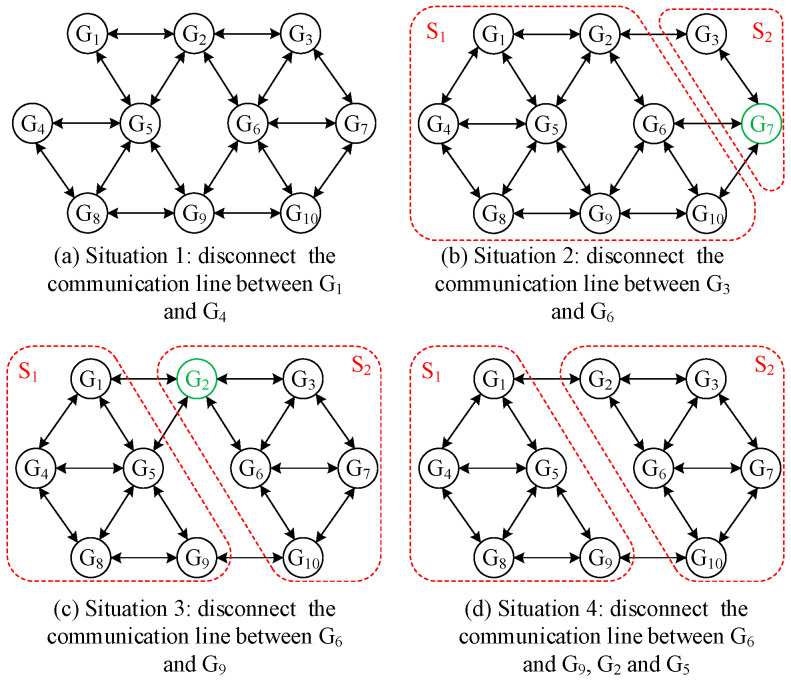
Different communication network topologies.

Definition 10 suggests that the communication network topology (Figure 4) is (2, 2)-robust. The simulation results in Section 5.2 also show that the economic dispatch system with the W-MSR algorithm under this topology can effectively resist various attacks. To verify the correctness of Theorem 1, some communication lines are disconnected to obtain various topologies, as shown in Figure 10.

Situation 1: Disconnect the communication line between G_1_ and G_4_, and the obtained communication topology is still a (2, 2)-robust graph.

Situation 2: Disconnect the communication line between G_3_ and G_6_. Subsequently, two subsets, namely, S_1_ and S_2_, can be obtained, as shown in Figure 10b. None of the conditions in Formula (19) is satisfied since $\left|X_{S_{1}}^{2}\right|=0$ and $\left|X_{S_{2}}^{2}\right|=1$ (node 7). Thus, the communication network topology is not a (2, 2)-robust graph at this time.

Situation 3: Disconnect the communication line between G_6_ and G_9_. As shown in Figure 10c, two subsets, namely, S_1_ and S_2_, can be obtained. These two subsets do not meet any of the conditions in Formula (19). Therefore, the communication network topology at the moment is still not a (2, 2)-robust graph.

Situation 4: The communication between G_2_ and G_5_ is further disconnected on the basis of Situation 3. It can be learned from Figure 10d that $\left|X_{S_{1}}^{2}\right|=0$ and $\left|X_{S_{2}}^{2}\right|=0$, and the topology connectivity has deteriorated further.

**Figure 11:**
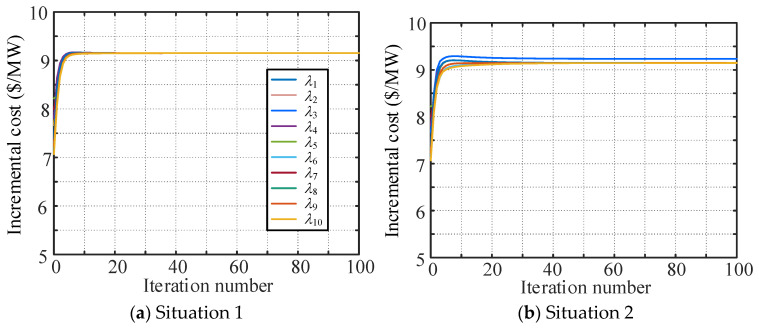

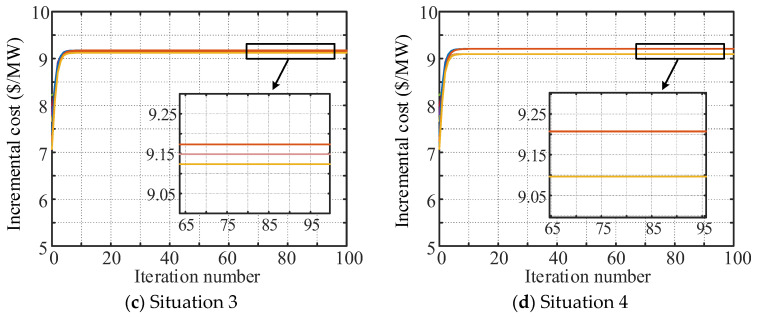
Convergences of the consensus variables under different communication network topologies.

Under the four communication network topologies, the convergences of the consensus variables when adopting the W-MSR algorithm with parameter *F* = 1 are shown in Figure 11.

Figure 11a shows that when the communication topology graph is still (2, 2)-robust, the distributed economic dispatch system can converge to operate at the optimal solution. Figure 11b–d show that when the communication topology is not a (2, 2)-robust graph, the system cannot converge to a consistent value. In comparison with Situation 3, the topological conditions of Situation 4 are worse, and the differences of the final consensus variables are larger. In fact, the topological graphs of communication networks under the four situations are also strongly connected, and their vertex connectivity is 2, 2, 3, and 3, respectively. Therefore, it can be concluded that using traditional network connectivity to judge the convergence of a distributed control system is not suitable for the distributed secure economic dispatch system, while the network robustness indicator is a more qualified substitute for a communication network that has a switching topology “induced”. In addition, the condition that the communication network graph *G* is an (*F* + 1, *F* + 1)-robust graph is the sufficient and necessary condition for the distributed secure economic dispatch to achieve its dispatch goal under information attacks.

## 6. Conclusions

This paper focuses on the information security of distributed economic dispatch systems. Considering that the distributed dispatch mode is vulnerable to external attacks and the identification of attacks in the decentralized structure is difficult, a distributed security economic dispatch strategy based on the W-MSR algorithm is proposed. The information that may come from the nodes under several attack modes is filtered out while updating the consensus variable of normal nodes. This strategy is performed by taking the incremental cost of the power generation unit as the consensus variable to realize a safe and economic dispatch. For the switching of communication network topology induced by the W-MSR algorithm, the conditions to be met for the communication network topology of the system are provided on the basis of the network robustness index. Theoretical analysis and simulation results suggest the following:

(1) The distributed dispatch strategy designed in this paper, based on the W-MSR algorithm, can withstand various modes of node information attacks. It ensures that the system re-establishes power balance and operates stably at the optimal value after suffering node attacks, effectively enhancing both the security and economic efficiency of the distributed economic dispatch system.
(2) The robustness of the network is suitable for measuring the performance of the communication network with an induced switching topology.
(3) The condition that the communication network topology of the distributed economic dispatch system is an (*F* + 1, *F* + 1)-robust graph, which is the necessary and sufficient condition for implementing the W-MSR algorithm, provides a basis for designing a communication network for a distributed secure and economic dispatch system.

Although the strategy proposed in this paper effectively enhances the security and economic efficiency of distributed systems, future research can further advance and deepen the following aspects:

This paper assumes that the number of attacked nodes and the patterns of information attacks are known. However, in real-world scenarios, attack behaviors may be more complex and difficult to predict. Future research could explore machine learning-based attack detection methods to address unknown attack patterns.

## Figures and Tables

**Table 1:** Parameter values of the generators.

Unit	*a_i_* (USD/MW^2^)	*b_i_* (USD/MW)	*c_i_* (USD)	$P_{Gi}^{\min}$ (MW)	$P_{Gi}^{\max}$ (MW)
G_1_	0.0038	6.87	135.88	75	500
G_2_	0.0034	7.03	214.92	80	400
G_3_	0.0029	7.58	108.23	30	280
G_4_	0.0018	7.67	220.00	80	420
G_5_	0.0016	8.14	232.56	50	350
G_6_	0.0025	7.15	78.09	50	480
G_7_	0.0022	7.86	234.48	64	300
G_8_	0.0026	6.80	74.60	45	500
G_9_	0.0033	5.99	127.69	74	400
G_10_	0.0028	6.65	100.52	150	600
G_11_	0.0040	6.32	128.36	80	400
G_12_	0.0028	6.79	187.63	90	350
G_13_	0.0033	6.54	254.23	75	450
G_14_	0.0022	8.97	98.76	40	500
G_15_	0.0018	7.32	118.45	45	300
G_16_	0.0041	8.64	186.34	30	420
G_17_	0.0035	7.51	225.79	65	600
G_18_	0.0021	8.28	178.69	30	350
G_19_	0.0024	6.97	169.24	50	480
G_20_	0.0027	8.16	169.31	60	650

**Table 2:** Key system information under Crashed attack.

Scenario	Consensus Variable (USD/MW)	Power of Each Unit (MW)	Power Imbalance (MW)
Consensus algorithm + no attack	[9.152, 9.152, 9.152, 9.152, 9.152, 9.152, 9.152, 9.152, 9.152, 9.152]	[300.285, 312.083, 271.063, 411.713, 316.302, 400.433, 293.674, 360.032, 327.601, 446.815]	0
Consensus algorithm + Crashed attack	[8.576, 8.558, 8.576, 9.051, 8.828, 8.828, 9.051, 9.204, 9.187, 9.204]	[224.448, 224.768, 171.691, 383.697, 214.949, 335.567, 270.752, 370.003, 332.824, 456.075]	455
W-MSR algorithm + Crashed attack	[9.152, 9.152, 9.152, 9.152, 9.152, 9.152, 9.152, 9.152, 9.152, 9.152]	[300.285, 312.083, 271.063, 411.713, 316.302, 400.433, 293.674, 360.032, 327.601, 446.815]	0

**Table 3:** Key system information under Crashed attack.

Scenario	Consensus Variable ($/MW)	Power of Each Unit (MW)	Power Imbalance (MW)
Consensus algorithm + no attack	[8.329, 8.329, 8.329, 8.329, 8.329, 8.329, 8.329, 8.329, 8.329, 8.329, 8.329, 8.329, 8.329, 8.329, 8.329, 8.329, 8.329, 8.329, 8.329, 8.329]	[192.038, 191.102, 129.223, 183.192, 59.216, 235.898, 106.702, 201.825, 202.953, 299.909, 251.186, 274.909, 271.135, 40.000, 280.414, 30.000, 117.070, 30.000, 283.227, 60.000]	0
Consensus algorithm + Crashed attack	[7.892, 8.013, 7.902, 8.203, 8.046, 8.057, 8.223, 8.374, 8.345, 8.301, 8.372, 8.407, 8.450, 8.465, 8.482, 8.501, 8.504, 8.559, 8.585, 8.590]	[134.427, 144.579, 55.578, 148.020, 50.000, 181.483, 82.587, 210.374, 205.266, 294.891, 256.444, 288.731, 289.386, 40.000, 300.000, 30.000, 141.998, 66.537, 336.520, 79.628]	103.55
W-MSR algorithm + Crashed attack	[8.329, 8.329, 8.329, 8.329, 8.329, 8.329, 8.329, 8.329, 8.329, 8.329, 8.329, 8.329, 8.329, 8.329, 8.329, 8.329, 8.329, 8.329, 8.329, 8.329]	[192.038, 191.102, 129.223, 183.192, 59.216, 235.898, 106.702, 201.825, 202.953, 299.909, 251.186, 274.909, 271.135, 40.000, 280.414, 30.000, 117.070, 30.000, 283.227, 60.000]	0

**Table 4:** Number of iterations required for system recovery.

Attack	10 Distributed Generation Units	20 Distributed Generation Units
Crashed attack	16	171
Byzantine attack	23	31
	27	46

## Data Availability

The data that support the findings of this study are available from the corresponding author upon reasonable request.
